# The analgesic efficacy of bilateral superficial cervical plexus block for thyroid surgery under general anesthesia: a prospective cohort study

**DOI:** 10.1186/s13104-020-4907-7

**Published:** 2020-01-28

**Authors:** Yophtahe B. Woldegerima, Amare G. Hailekiros, Girmay L. Fitiwi

**Affiliations:** Department of Anesthesia, College of Medicine and Health Sciences, University of Gondar, Gondar, 196 Ethiopia

**Keywords:** Bilateral superficial cervical plexus block, Postoperative pain, Thyroidectomy, Cervical plexus, Multimodal analgesia

## Abstract

**Objective:**

Uses of simple analgesics were found insufficient to manage pain after thyroid surgery. We hypothesized that using bilateral superficial cervical plexus block (BSCPB) might influence the pattern of immediate postoperative pain and analgesic consumption. The general objective of the study was to assess the analgesic efficacy of bilateral superficial plexus block for thyroid surgery under general anesthesia.

**Results:**

A total of 74 willing patients involved. Half of them had received BSCPB with 10 ml of 0.25% bupivacaine just before induction and the remaining half did not. Postoperatively, patients were assessed at immediate, 2nd, 6th, 12th and 24th h. At all endpoints, NRS-11 scores for pain were significantly lower in the block group. The time to first analgesic requirement was significantly longer 132.3 ± 71.5 min vs 71.4 ± 60.0 min, p = 0.009. Opioid and total analgesic consumption were reduced by BSCPB in the first 24 postoperative hours. There was low but non-significant rate of PONV in the block group. No clinically important adverse event was noted related to BSCPB.

*Trial registration* The study was registered in Pan African Clinical Trial Registry on 13/01/2020 and the registration number is PACTR202001579588451. Retrospectively registered.

## Introduction

Thyroid disease is one of the major public health problems in Ethiopia [1–3]. Hence, thyroid surgery is one of the frequently performed surgical procedures. It is also the leading endocrine surgery world-wide [4]. The prevalence of goiter among school children in Ethiopia was estimated between 20 and 50% and even more. It was explained by iodine deficiency [1–3]. Despite this huge implication and frequency of thyroid surgery, the analgesic efficacy of bilateral superficial plexus block for thyroid surgery was not determined in our population. In addition to the differences in the patterns of thyroid diseases in our population, it’s strongly suggested that pain severity and perception have significant differences among populations worldwide [5]. Most of previously published studies were done in different population. Furthermore, the surgical and anesthetic service settings in Africa have big differences compared to the developed world including interpersonal and technical differences. The developing world has many barriers for appropriate management of acute pain in where only 7% of opioid consumed from the global consumption [5].

Acute pain is one of the commonest complaints in the postoperative period which has serious adverse cardiovascular, pulmonary, metabolic and psychological outcomes [5–8]. Pain after thyroid surgery is significant especially in the early postoperative hours. The mean score of post-thyroidectomy pain was 6.9 ± 1.7 on visual analog scale and 90% of patients required morphine [9]. Despite paracetamol administration, 70% of patients initially rate a score ≥ 4 on numeric rating scale (NRS-11) [10]. Recently, a study has shown that 93% of patients required up to 20 oral morphine equivalents [4].

Simple analgesics such as paracetamol and non-steroidal anti-inflammatory drugs (NSAIDs) were found insufficient to manage pain after thyroidectomy [11]. Bilateral superficial cervical plexus block (BSCPB) is widely used for managing pain after thyroid surgery. Studies reported that the block allowed to reduce anesthetic requirements and provided prolonged postoperative analgesia. It also decreased pain score, rescue analgesic requirement and overall opioid requirement in the first 24 postoperative hours [12–14]. Hence, it minimizes opioids related adverse outcomes and cost [15]. BSCPB was found simple, safe, cheap and effective for post-thyroidectomy pain management [16]. However, ineffectiveness of BSCPB was also reported [17–19].

We hypothesized that using BSCPB may influence the pattern of immediate postoperative pain and analgesic consumption. The general objective of the study was to assess the analgesic efficacy of bilateral superficial plexus block for thyroid surgery under general anesthesia.

## Main text

### Methodology

#### Study design, area, period and population

A prospective cohort study was conducted in University of Gondar Hospital (UoGH), Northwest Ethiopia from February to June 2016. The study was registered in Pan African Clinical Trial Registry (PACTR202001579588451). All adult (18+) ASA I and II patients who undergone thyroidectomy at the hospital during the study period were included in the study. Patients who have refused to participate, allergic history for local anesthetics, retro-sternal goiter, altered anatomical landmarks, coagulation abnormality and other contraindications for the block were excluded.

#### Variables

Pain severity, time to first analgesic request and total 24 h analgesics consumption were outcome variables. Socio-demographic, ASA class, size and type of thyroid mass and duration of anesthesia and surgery were some of the independent variables.

#### Sample size determination and sampling technique

The sample size was determined by postoperative morphine requirement in the first 24 h. BSCPB with 0.25% of bupivacaine reduced morphine requirement (mg/Kg) in the first 24 postoperative hours by nearly 55% median in mg/Kg (0.38 Vs 0.69, p = 0.01) [20]. Calculation was done using predetermined 5% margin of error (α), and power of 80% (β), ∫ (α, β) is 7.85.

$$ \begin{aligned} {\text{Patients}}\;{\text{per}}\;{\text{group}} & = \frac{{x1\left( {1 - x1} \right) + x2\left( {1 - x2} \right)}}{{\left( {x1 - x2} \right)^{2} }} \times \smallint \left( {\alpha  \beta } \right) \\ & = \frac{{ 0.69\left( {1 - 0.69} \right) + 0.38\left( {1 - 0.38} \right)}}{{\left( {0.69 - 0.38} \right)^{2} }} \times 7.85 \\ & = \,36.7\, \approx \,37\,{\text{patients}}\;{\text{in}}\;{\text{each}}\;{\text{group}} \\ \end{aligned} $$

The patients in the block group had received BSCPB with 10 ml of 0.25% bupivacaine on each side along the posterior border of sternocleidomastoid (from the midpoint 2 ml to caphal 4 ml and caudal 4 ml) immediately before induction. The patients in non-block group did not receive BSCPB and placebo injection of normal saline was not done.

#### Data collection, quality control and analysis

Two data collectors were assigned. One for pre- and intraoperative time, and another for postoperative time to facilitate blinding. Assessments were done at postoperatively in the recovery room; immediately after arrival, 2nd, 6th, 12th and 24th h. Postoperative pain was assessed using NRS-11. First analgesia request time, and total analgesia consumption within 24 h were documented. Data was checked and analyzed by SPSS-20 (IBM Corporation). Normality was checked by Shapiro–Wilk test. An independent t-test was performed to compare time to first analgesic request. Mann–Whitney U test was used to analyze repeated NRS-11 scores and total postoperative analgesic consumption. Normally distributed data was presented in mean ± SD whereas non-normally distributed data was presented as median (IQR). A p-value < 0.05 was considered as statistically significant.

## Results

### Demographic, anthropometric and clinical characteristics of participants

A total of 74 patients (34 in each group) were involved. The demographic, anthropometric and clinical characteristics of participants were found comparable between the groups (Table 1). Sub-total and near total thyroidectomy were the leading types of thyroid surgery in the block group and non-block group respectively. The length of incision was 9.2 ± 2.8 vs 9.1 ± 2.1 in the block group and non-block group respectively and no statistically significant difference between the groups. Simple nodular goiter was the most frequent (22; 59.4%) diagnosis in the block group and multi-nodular goiter in the non-block group (16; 43.2%). Only 4 patients (1 in block group and 3 in non-block group) had undergone extended neck dissection. The use of preemptive analgesia with simple analgesics and opioids was comparable. There was no difference in choices of induction agents. The larger proportions of patients in both groups were induced with propofol (block group = 75.7% vs non-block group = 67.6%, p > 0.05) and the remaining with thiopentone.

**Table 1:** Demographic and clinical characteristics of patients, frequency and percentage (n (%)) from Chi square test, mean ± standard deviation from independent t-test, N = 74

Variables	Block group (n = 37)	Non-block group (n = 37)	p-value
Age (years)	35.1 ± 9.3	34.6 ± 10.0	0.85
BMI	20.1 ± 2.4	20.4 ± 3.3	0.74
Sex			0.30
Male	10 (27)	6 (16.3)	
Female	27 (73)	31 (83.7)	
ASA class			0.99
I	30 (81)	30 (81)	
II	7 (19)	7 (19)	
Diagnosis			0.63
Simple nodular goiter	22 (59.4)	12 (32.4)	
Simple colloid goiter	6 (16.2)	6 (16.2)	
Multi-nodular goiter	8 (21.6)	16 (43.2)	
Thyroid cancer	1 (2.7)	3 (8.1)	
Size of thyroid mass (cm^2^)	31.8 ± 24.2	37.5 ± 26.2	0.43
Type of thyroidectomy			0.12
Lobectomy	0 (0)	0 (0)	
Subtotal	22 (59.4)	12 (32.4)	
Near total	12 (32.4)	21 (56.7)	
Total	2 (5.4)	1 (2.7)	
Extended neck dissection	1 (2.7)	3 (8.1)	
Incision length (cm)	9.2 ± 2.8	9.1 ± 2.1	0.96
Duration of surgery (min)	120.2 ± 36.6	123.4 ± 41.4	0.77
Duration of anesthesia	140.4 ± 38.0	145.6 ± 43.8	0.66
Preemptive analgesia at induction			0.57
Acetaminophen and diclofenac	4 (16)	3 (12)	
Acetaminophen, diclofenac and opioids	21 (84)	22 (88)	

### Patterns of pain and analgesic requirements

At all endpoints, pain scores were significantly lower in the block group. Furthermore, the first analgesic request time was significantly longer in the block group than the non-block group (Table 2). The total analgesic consumption in the first 24 postoperative hours was significantly reduced in the group that received BSCPB. Surprisingly, none of patients in the block group required strong opioid analgesics. However, 24 h pethidine consumption was 34 ± 15.1 mg in the non-block group (Table 3).

**Table 2:** Postoperative numeric rating scale-11 pain scores: median (IQR), and first analgesic request time: mean ± standard deviation from Mann–Whitney U-test

Group	Block group (n = 37)	Non-block group (n = 37)	p-value
NRS-11 at immediate postoperative time	0 (5)	6 (4)	0.001
NRS-11 at 2nd h	2 (6)	7 (2)	< 0.001
NRS-11 at 6th h	2 (4)	5 (3)	0.001
NRS-11 at 12th h	0 (3)	4 (3)	< 0.001
NRS-11 at 24th h	0 (1)	3 (3)	< 0.001
First analgesic request time (min)^a^	132.3 ± 71.5	71.4 ± 60.0	0.009

N = 74

^a^Independent t-test

**Table 3:** Total postoperative analgesic consumption: mean ± standard deviation from Mann–Whitney U-test

Group	Block group (n = 37)	Non-block group (n = 37)	p-value
Diclofenac (mg)	75 ± 0	82 ± 24.2	0.003
Tramadol (mg)	90 ± 22.4	104.55 ± 37.5	0.004
Pethidine (mg)	0	34 ± 15.1	0.001

N = 74

## Discussion

We found statistically significant reduction in mean NRS-11 scores at all end-points in the block group. The time to first analgesic requirement was nearly doubled in the block group (132.3 ± 71.5 min vs 71.4 ± 60.0 min, p = 0.009). Multiple studies have investigated the effectiveness of BSCPB in thyroid surgery and reported that it was effective in minimizing pain scores, opioid and total analgesic consumption and prolonging analgesia duration [12, 13, 21, 22]. A meta-analysis of 14 studies incorporated 1154 patients revealed BSCPB significantly reduced analgesic requirement, VAS scores and lengthen time to first analgesic request [14]. BSCPB was found significantly associated with nearly half shorter postoperative hospital staying days (2.4 ± 0.6 vs 4.7 ± 1.6; p < 0.05) [12].

In-contrast, some studies denied the effectiveness of BSCPB. The block had failed to demonstrate reduction in pain scores and opioid consumption. But longer time for first analgesic request was observed. They explained the result by pain arising from deeper and muscular structures, pain from positioning and wound drainages [17]. Despite these, pain after thyroidectomy was known to have large superficial component [23]. Different drug regimens, volumes, techniques of injections and duration of postoperative follow-up (36 h) might be possible causes for these contradictory conclusions [18]. Another study has concluded equi-vocal as BSCPB reduced pain intensity and analgesic requirement but could not provide optimal pain relief alone since 65% of patients need additional analgesia [10]. Performing the block after the surgery might have effect on this equi-vocal outcome. In another study, hospital stay and postoperative analgesic consumption were comparable even if patients in the block group had lesser VAS scores. These differences might be due to 4 days follow-up [24].

In this study, all blocks were done by landmark technique by subcutaneous deposition of local anesthetic along the posterior borders of sternocleidomastoid muscles on both sides of the neck. In a recent Egyptian study that compared landmark and ultrasound-guided techniques found no difference in effectiveness and safety [25]. However, another study has concluded that an ultrasound-guided technique had superiority and explained by direct visualization of the nerves, adjacent structures and needle movement that results in faster, denser and longer block [26].

Performing regional nerve blocks and administration of multi-modal analgesics prior to surgical incision are helpful in reducing intra- and postoperative opioid consumption, primary hyperalgesia, central sensitization and chronic pain [14, 23, 27]. In combination with gabapentin, BSCPB has prevented delayed neuropathic pain at 6th postoperative month [28]. Thyroidectomy without BSCPB was three-times likely associated with neuropathic pain compared to thyroidectomy with BSCPB [29]. In our study, all BSCPBs were done in the preoperative time, immediately before induction as a part of multi-modal analgesia. This might provide the benefits of preemptive analgesia and minimized anesthetic duration. Some surgeons complained for disruption of the surgical anatomy by the block. In another study, according to surgeon’s opinions, the surgical conditions were very good and had encountered no problem [23]. An ultrasound-guided study suggested that performing BSCPB in the pre- or postoperative time were equally effective. Landmark technique was also found effective whether performed in the pre- or postsurgical time to reduce the VAS scores [24]. Furthermore, presurgical block is technically easier unless in very large thyroid mass. After surgery, anatomical planes may be changed and facilitate leakage through incision and facial layers [13]. However, Herbland and colleagues reported that irrespective of time of injection (pre- or postsurgical), BSCPB is not effective analgesic option for thyroidectomy. They explained it by incomplete sensory block because of limited spread of solution through the investing fascia and high vascularity of the area [18].

Wound infiltration is effective choice of analgesia after thyroid surgery. But compared to BSCPB, the later was found more effective. Time to first analgesia were 162 ± 124 min vs 544 ± 320 min vs 860 ± 59 min in control, wound infiltration and BSCPB groups respectively; p < 0.001 [30]. This analgesic duration was very long compared to our finding. This difference might be due to drug regimen as they used 15 ml of 0.5% bupivacaine and in the current study 10 ml of 0.25% bupivacaine. Two recent RCTs have declared that wound infiltration lacks effectiveness for treating pain after thyroidectomy; even in addition of adrenaline [31, 32].

The incidences of postoperative nausea and vomiting (PONV) after thyroidectomy ranges from 21.7% up to 84% [12, 33]. We have assessed PONV with simplified PONV impact scale and the incidence of clinically important PONV was 27% in block group and 35.1% in non-block group but no statistically significant difference was observed. These results were lower compared to other studies. The reason might be predominant use of propofol for induction of anesthesia in the current study [18]. Despite lower incidences of PONV, we found that comparable between the groups. This phenomenon might be explained by tramadol consumption. Even though, there was statistically significant reduction in tramadol consumption, patients in the block group might have consumed clinically significant amount of tramadol. No clinically significant complication occurred in association with BSCPB.

We have concluded that BSCPB has significantly reduced pain scores, opioid and total analgesic consumption and prolong the time to first analgesic requirement. We recommend that BSCPB is simple and can be used effectively and safely for pain management after thyroid surgery as a part of multi-modal analgesia in the first 24 postoperative hours.

## Limitations

As a cohort study, the confounders might not be adequately controlled. We also have not studied the impact of the block on intraoperative analgesic and anesthetic requirements.

## Data Availability

Data and materials used in this study are available and can be presented by the corresponding author upon reasonable request.

## Abbreviations

ASA: American society of anesthesiologists
BSCPB: bilateral superficial cervical plexus block
IBM: International Business Machines
IQR: interquartile range
NRS-11: 11-points numeric rating scale
NSAIDs: non-steroidal anti-inflammatory drugs
PONV: postoperative nausea and vomiting
RCTs: randomized control trials
SPSS-20: statistical package for social studies 20th version
SD: standard deviation
UoGH: University of Gondar Hospital
